# Fast Semantic Segmentation of Remote Sensing Images Using a Network That Integrates Global and Local Information

**DOI:** 10.3390/s23115310

**Published:** 2023-06-03

**Authors:** Boyang Wu, Jianyong Cui, Wenkai Cui, Yirong Yuan, Xiancong Ren

**Affiliations:** College of Oceanography and Space Informatics, China University of Petroleum (East China), Qingdao 266580, China; 2016020121@s.upc.edu.cn (B.W.); 1916020106@s.upc.edu.cn (W.C.); 2016020124@s.upc.edu.cn (Y.Y.); 2016040119@s.upc.edu.cn (X.R.)

**Keywords:** remote sensing images, semantic segmentation, global and local information

## Abstract

Efficient processing of ultra-high-resolution images is increasingly sought after with the continuous advancement of photography and sensor technology. However, the semantic segmentation of remote sensing images lacks a satisfactory solution to optimize GPU memory utilization and the feature extraction speed. To tackle this challenge, Chen et al. introduced GLNet, a network designed to strike a better balance between GPU memory usage and segmentation accuracy when processing high-resolution images. Building upon GLNet and PFNet, our proposed method, Fast-GLNet, further enhances the feature fusion and segmentation processes. It incorporates the double feature pyramid aggregation (DFPA) module and IFS module for local and global branches, respectively, resulting in superior feature maps and optimized segmentation speed. Extensive experimentation demonstrates that Fast-GLNet achieves faster semantic segmentation while maintaining segmentation quality. Additionally, it effectively optimizes GPU memory utilization. For example, compared to GLNet, Fast-GLNet’s mIoU on the Deepglobe dataset increased from 71.6% to 72.1%, and GPU memory usage decreased from 1865 MB to 1639 MB. Notably, Fast-GLNet surpasses existing general-purpose methods, offering a superior trade-off between speed and accuracy in semantic segmentation.

## 1. Introduction

The advancement of computer science and sensor technology has led to the extensive use of remote sensing in various fields, such as environmental science, ecology, and urban planning [1,2,3,4,5,6]. Obtaining spatial and attribute information from remote sensing images plays a crucial role in the progress of remote sensing. In this context, semantic segmentation emerges as a vital technique for interpreting remote-sensing images, forming an integral part of computer vision. It serves as a significant method for processing and analyzing both low-level and high-level remote sensing images, enabling pixel-level object categorization.

Semantic segmentation finds applications in diverse sectors, including autonomous vehicles, medical imaging and diagnostics, and UAV landing site selection. As photography and sensor technology continues to develop, there is an increasing demand for processing ultra-high-resolution images. High-resolution remote sensing images contain complex information about ground objects. However, traditional segmentation methods do not adequately consider the semantic content of remote-sensing images, resulting in suboptimal segmentation results. Consequently, the application of conventional image segmentation approaches is limited, necessitating the development of new methods to address these challenges.

Deep learning, a critical subfield of machine learning, involves training multilayer neural networks to perform classification or regression tasks on unclassified data, which has gained prominence in several domains, including computer vision, thanks to technology such as big data and high-performance computation. Convolutional neural networks have gained significant attention as a type of deep learning network. Multiple hierarchical layers, including input, convolutional, pooling, and fully connected layers, make up a convolutional neural network. Deep learning offers the advantage of replacing manual feature extraction with unsupervised or weakly supervised feature learning, along with hierarchical feature extraction algorithms. Because the features extracted by CNNs will increase with visual characteristics, deep learning models can be used as feature extraction tools with the function of extracting high-level features of images. Currently, deep learning methods are mainly used in feature extraction and image matching. As more in-depth studies on deep learning have been conducted recently, especially those on CNNs used for feature extraction, great breakthroughs have been made in semantic segmentation methods represented by deep convolutional neural networks in many fields.

The purpose of remote sensing images object recognition is highly consistent with the function of semantic segmentation, and deep learning technology has a strong ability with respect to nonlinear expressions. For these reasons, the coupling of deep learning and the process of object recognition from remote sensing images has emerged as a predominant research direction. The introduction of fully convolutional networks (FCNs) [7] marked the beginning of the FCN era in this field. The advent of FCNs led to the development of several cutting-edge semantic segmentation models based on deep learning. Examples include U-Net [8], SegNet [9], PSPNet [10], RefineNet [11], and others [12,13,14,15,16,17,18].

In recent years, many excellent semantic segmentation networks for remote sensing image processing have emerged. MANet [19] adopts a novel dual-attention mechanism and a new cross-dimensional interactive attention feature fusion module, which enhances feature extraction and fusion. Based on U-Net, FPUA [20] uses a feature pyramid module combined with an attention structure to improve the segmentation accuracy of the network for small objects. To enhance the regional integrity of images and thus reduce misclassification, RE-Net [21] introduces the regional information into the base network. Combining PCA, the attention mechanism, and U-Net, PSE-UNet [22] analyzes the factors affecting the performance, which makes it superior to other semantic segmentation algorithms. KGGCN [23] uses superpixel blocks as graph network nodes, combines prior knowledge with spatial correlation in the process of information aggregation, and effectively overcomes the distortion of sample context. MCSGNet is a semantic segmentation network for classifying land-use types in remote sensing images. To improve the efficiency of segmentation, they proposed the RCA module and the CAII module. The SIC module is used to realize the information exchange between different layers. MCSGNet has good segmentation accuracy and generalization ability, which has good segmentation performance on the LandCover dataset, with an mIoU reaching 87.432%. BiSeNet [24], a cutting-edge bilateral segmentation network, was developed to effectively maintain spatial location information and produce high-resolution feature maps through the implementation of a spatial path with minimal steps. Simultaneously, a semantic path featuring a rapid downsampling rate was designed to acquire the target receptive field. Furthermore, a novel feature fusion module was introduced to merge the feature graphs of both modules, thereby achieving an optimal balance between speed and accuracy. Rudra et al. combined the classical encoder–decoder framework [7,9] and multibranch framework [24,25,26] to propose a new semantic segmentation architecture: Fast-SCNN [27]. Based on the existing semantic segmentation of two branches, the “learning to downsample” module is used to compute the low-level features of multiple resolution branches simultaneously. The model achieved an mIoU of 68.0% on the Cityscapes datasets for high-resolution (1024 × 2048) images, as demonstrated by tests conducted on an NVIDIA Titan XP GPU.

With the development of photography and sensor technology, the demand for processing ultra-high-resolution images is growing. There has been no effective method offered to cope with the challenges of GPU memory utilization and feature extraction speed in high-resolution image semantic segmentation.

Regarding the optimal use of GPU memory, Chen et al. proposed a network named GLNet [28]. With the ResNet [29] and FPN (the feature pyramid network) [30] as the backbones of its two branches, GLNet can effectively balance the use of GPU memory and the accuracy of semantic segmentation. CascadePSP [31] uses the global step and the local step to perform high-resolution segmentation refinement. Experimental results show that using both steps results in lower GPU memory usage compared to using the global step only. The difference is approximately 4% mBA. Wu et al. introduced the guided filtering layer [32], which was designed to efficiently generate high-resolution output from a corresponding low-resolution input and a high-resolution guidance map. Their models on the MIT-Adobe FiveK dataset show faster speed and higher performance than other models. MagNet [33] consists of multiple processing stages, each corresponding to a level of amplification. The output of one stage is fed into the next for coarse-to-fine information propagation. Each stage analyzes the image at a higher resolution than the previous stage, recovering details that were lost due to the downsampling step. The segmented output is progressively refined through each processing stage, resulting in a more accurate segmentation. MagNet exhibited a good performance in three challenging high-resolution image datasets of urban views, aerial scenes, and medical images.

In an effort to enhance both the computational speed and accuracy of semantic segmentation networks, Li et al. proposed a PointFlow module (PFM) [34] based on an FPN and designed a dual point matcher to address the issue of foreground–background imbalance and better balance the segmentation speed and accuracy.

We established a novel semantic segmentation network, named Fast-GLNet, which is built upon GLNet and PFM, and added the DFPA and IFS modules after integrating global and local branches. The Fast-GLNet, which combines the advantages of GLNet and PointFlow, can optimize the use of GPU memory while balancing the speed and accuracy of semantic segmentation.

In the experimental section, Fast-GLNet is studied and analyzed in detail, and its performance is compared with those of many other semantic segmentation networks. Additionally, some mainstream semantic segmentation networks and our Fast-GLNet were tested on the Vahihigen, Potsdam, and DeepGlobe datasets. It was found that Fast-GLNet achieved the best results on these benchmark datasets, achieving an optimal trade-off between speed and segmentation accuracy. The speed and precision of semantic segmentation may be more effectively balanced using this technique than with the current general-purpose methods.

## 2. Related Work

Traditional semantic segmentation techniques may be loosely divided into unsupervised learning techniques and probabilistic graphical model-based techniques. The effectiveness of feature selection has an impact on how well these algorithms function. Segmentation methods based on PGMs include random decision forests (RDFs) [35] and conditional random fields (CRFs). Unsupervised learning methods, such as K-means and mean-shift algorithms, are frequently used in semantic segmentation. Traditional approaches analyze high-resolution remote sensing images quickly, but they often perform poorly in semantic segmentation. Deep learning methods have achieved remarkable performance in image processing tasks as a result of the growth of huge datasets and hardware computing power, and semantic segmentation methods based on deep learning are particularly useful in practical applications.

The proposal of fully convolutional networks (FCNs) [7] for pixel-level prediction marked the inception of deep learning-based semantic segmentation methods. Deeplab [36], based on FCN [7], utilizes dilated convolution to widen the receptive field and optimizes feature extraction in order to obtain more feature information. Badrinarayanan et al. modified the VGG-16 [37] network based on an FCN and established a new semantic segmentation network known as Segnet [9] using the encoder–decoder architecture. RefineNet [11] improves the decoder structure and uses residual connections to achieve better fusion with high-level semantic features. UNet [8] adopts a symmetric encoder–decoder architecture with downsampling in the first half and upsampling in the second half.

Performance and speed problems are common in these networks. With the advancement of photography and sensor technology, the demand for processing ultra-high-resolution images is growing continuously. Compared with the segmentation of traditional images, the segmentation of geospatial objects in remote sensing images is more challenging. The use of GPU memory and the optimization of the feature extraction speed are not fully considered.

FarSeg [38] tackles the foreground–background imbalance problem in remote sensing images by effectively learning the contextual information of the foreground-scene relationship, thereby improving the detection of foreground features.

A featurized image pyramid extracts the features in each layer by means of image downsampling and performs prediction for each layer. The features are calculated independently depending on the size of each image, which will result in a high computational load, long reasoning time, and low efficiency.

The method of prediction using a single high-level feature map performs prediction through the last feature map, which was generated by the backbone. The RPN layer in Faster R-CNN classifies objects and performs bounding box regression using a single high-level feature map. As a result, the performance of this method is unsatisfactory in detecting small targets.

The pyramidal feature hierarchy is used to predict different feature maps, which are generated by the backbone and can better handle the multiscale problems with the targets to be processed. However, it tends to misclassify small targets because low-level feature maps lack semantic information.

To address the issues with the three structures mentioned above, the FPN [30] connects the feature maps from both shallow and deep layers, transmitting the information from the shallow layer to the deep layer to improve its prediction performance and solve the problem that the small targets of deep-layer feature maps tend to be neglected. Our fast fusion module uses a framework similar to the FPN, and an aggregation mechanism is incorporated into the decoder to optimize the prediction speed.

## 3. Method

### 3.1. Overview

**Figure 1:**
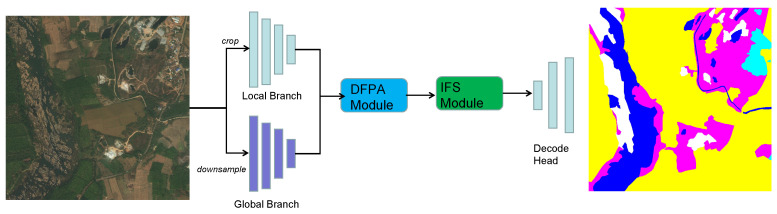
Overview of the Fast-GLNet. The local and global branches are composed of small images segmented from the original image and subsampled versions of the original image, respectively. After passing through the DFPA module and IFS module, the final semantic segmentation result is obtained.

The Figure 1 shows the overall framework of our model. The Fast-GLNet consists of global and local branches and can retain global information and details effectively. In addition, to obtain the ultimate segmentation outcome, the DFPA module and IFS module are used to deeply integrate the feature maps from the two branches.

### 3.2. Multibranch

Fast-GLNet consists of global and local branches. With the low-resolution global image obtained by downsampling as its input and ResNet [29] as its backbone, the global branch retains the global contextual information present in the original image. With the full-resolution cropped images as its input and an FPN [30] as its backbone, the local branch retains the details of the image. In addition, to facilitate training and testing, the method of ordered complete cropping was used instead of random cropping for local branch images. Each branch’s feature maps for every layer were extensively shared with the other branch. After aggregating the two groups of high-level feature maps, the branch aggregation layer was utilized to generate the final segmentation output. We used weak-coupling regularization for the local branch’s training to limit the two branches and ensure stable training. The DFPA module and IFS module were incorporated into Fast-GLNet after the integration of the two branches to improve the segmentation speed and segmentation performance.

To ensure better collaboration between the two branches, the global branch’s feature map was initially cropped at the current local patch’s position before being upsampled to the size of the local branch’s feature map. Then, they were connected as additional channels to the feature map from the local branch in the same layer. The feature map from the local branch was collected in a symmetrical manner. First, the local feature map was downsampled to match the position of the original image’s cropped patches. Next, the patches were merged (in the same order followed to crop local patches) into an entire feature map that was the same size as the global branch’s. Additionally linked to the channel for global feature maps, these local feature maps were transmitted to the following layer.

### 3.3. Double-Feature Pyramid Aggregation Module

**Figure 2:**
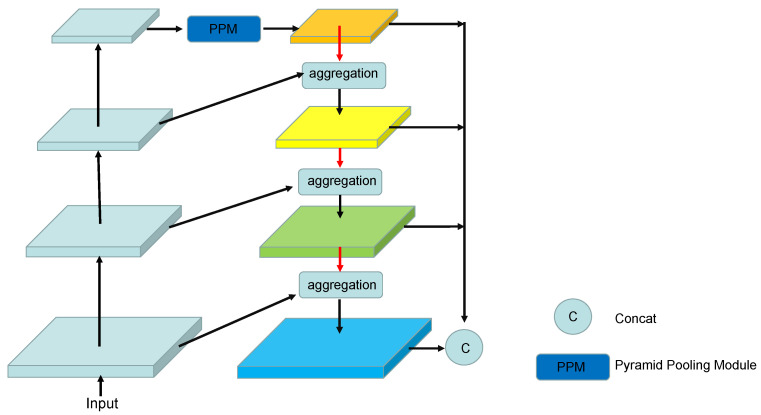
Double -feature pyramid aggregation module. We established the module architecture of the fast fusion module by incorporating an aggregation mechanism into a framework similar to an FPN [30]. The encoding part consists of a feature pyramid, and the decoding part is a lightweight FPN with an aggregation mechanism.

The double-feature pyramid aggregation module in Figure 2 consists of a bottom-up path that serves as an encoder and a top-down path that serves as a decoder, similar to an FPN. Multiple feature pyramids are output by the encoder’s backbone network. The aggregation mechanism is built into the decoder. The encoder of this network uses ImageNet as the pretraining backbone. In addition, the pyramid pooling module (PPM) [10] is used to obtain contextual information. In our design, we made sure that the output of the PPM matches the encoder’s final section’s resolution. The DFPA module decoder obtains the feature map from the encoder and achieves image segmentation through a redefined feature pyramid for image segmentation. To improve detection performance, bilinear upsampling, which is commonly used in the DFPA module, was replaced with an aggregation mechanism in order to connect all refined feature layers (where l ranges from 2 to 5) and perform predictions simultaneously by upsampling the input feature map to 1/4 of the input resolution. For edge prediction for each DFPA module, the binary cross-entropy expressed by Lbce is used as the loss function. The cross-entropy loss is used for the final segmentation prediction. The default weight of the two loss functions is 1.

**Figure 3:**
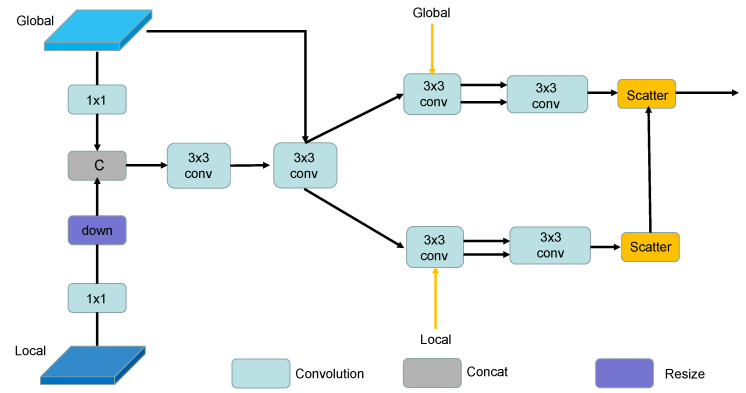
Overall aggregation pipeline. On the left side: perform a 1 × 1 convolution on the global and local branches, resize the local branch after the convolution, and concatenate the two branches for two 3 × 3 convolutions. On the right side: perform a 3 × 3 convolution on the convolution results on both sides with the two branches and then perform the dispersion operation.

Figure 3 illustrates our aggregation pipeline. For better collaboration with the local branch, a 1 × 1 convolution was performed on the two branches, and the local feature map was downsampled to match the global branch’s feature map’s size so that the two could be merged in the channel dimension to produce an entire feature map that was the same size as the global branch’s. Then, a 3 × 3 convolution was performed on the complete feature map, and the result of the convolution was used as the new local part. Moreover, a 3 × 3 convolution was performed again on the global branch, and the result of the convolution was used as the new global part. Subsequently, two 3 × 3 convolutions were performed on the new global and local parts, and the scatter operation was performed on the corresponding results to generate the final feature map.

## 4. Results

### 4.1. Dataset

The Vaihigen and Postdam datasets were used as the main datasets for our experiments.

The ISPRS provided two cutting-edge datasets consisting of aerial images for urban classification and 3D building reconstruction. These datasets utilize a digital surface model created from high-resolution orthogonal images and advanced image matching algorithms. Urban scenarios are included in both databases. Postdam is a perfect example of a classic old city, complete with massive structures, winding lanes, and densely packed habitations, while Vaihingen is a comparatively tiny town with numerous individual buildings and modest multistory buildings. Each dataset underwent manual classification into six land-cover categories. Water bodies and items that differ from other established classes are common backgrounds in metropolitan settings and are frequently semantic objects of indifference.

The 33 remote sensing images that make up the Vaihingen dataset are all various sizes and were all taken from a bigger top-level orthophoto. The image selection process was completed in such a way as to avoid data unavailability. The top-level picture and the digital surface model have a 9 cm spatial resolution. The remote sensing images are 8-bit TIFF files consisting of three wavebands. The digital surface model, which encodes the height information as 32-bit floating-point numbers, is a single-band TIFF file.

The Postdam dataset consists of 28 identically sized images. The top-level picture and the DSM have a 5 cm spatial resolution. This dataset, like the Vaihingen dataset, is made up of TIFF files with three wavebands and a digital surface model, with all images being the same size. There, the DSM and all remote sensing images are defined using the UTM WGS84 as the reference system. An affine transformation file is included for each image, allowing for the easy decomposition of the image into smaller subimages as necessary. These datasets also provide a means of TOP image storage in the TIFF format for different channel combinations so that participants can select the desired data.

The files include regular DSM and normalized DSM, which reflect height above the terrain by eliminating each pixel’s ground height following ground filtering. Multiple totally automatic filtering procedures create the data, with no manual quality control. Therefore, the absence of incorrect or false data cannot be guaranteed. The purpose of these datasets is to help researchers use high-level data instead of using the absolute DSM.

### 4.2. Experimental Details

We used the FPN [30] and ResNet50 [29] as the backbone networks in our work. The ResNet50’s feature maps in the bottom-up phases, and the FPN’s feature maps in the top-down and smoothing phases have deep-sharing. For the last horizontal connection in the FPN, we performed the regularization of feature maps and took the aggregation result at this stage as the final segmentation result. The same size (500 × 500) was used for both the cropped local patches and the downsampled global images for simplicity. Adjacent patches were overlapped by 50 pixels to prevent the boundaries of all convolution layers from vanishing. The primary loss and two auxiliary losses were optimized with the focal loss [39]γ = 6 as the goal. The primary and auxiliary losses were given the same weights (1.0). It was decided to use a feature-map regularization coefficient of 0.15.

We utilized the “gpustat” command-line tool with a minimum batch size of 1 to monitor the GPU memory consumption using a model while ensuring no gradient calculations were performed. It is to be noted that only one GPU was used for training and reasoning.

PyTorch [40] was used to conduct the experiments. To train the global branch and the local branch, we utilized Adam optimizers [41] with learning rates of $1\times 10^{-4}$ and $2\times 10^{-5}$, respectively. For all training sessions, a minibatch with the size of 6 was used. All of the tests were carried out on workstations using NVIDIA 1080Ti GPU cards.

### 4.3. Experimental Results

As illustrated in Table 1 and Table 2, some mainstream semantic segmentation networks and our Fast-GLNet were tested on the Vahihigen and Potsdam datasets. It has been found that, for the Vahihigen dataset, the mIoU values of PsPnet [10], FCN [7], and other networks were smaller than 67%, and only the mIoU values of GSCNN [42] and SFNet [43] were greater than 67%. In addition, the mIoU value of our Fast-GLNet even reached 73.2%. Only GSCNN’s mean-F1 value approached 80%, while Fast-GLNet’s was 82.7%. For the Potsdam dataset, the mIoU values of common networks were around 73% and 74%, and the mean-F1 values of these networks were about 83%. In comparison, the mIoU values of Fast-GLNet reached 77.9% and 86.1%. These favorable results delivered by Fast-GLNet are attributable to the mode of collaboration between the global and local branches, which can decrease the amount of GPU memory used and optimize the speed of semantic segmentation.

We also tested some mainstream semantic segmentation networks and our Fast-GLNet on the DeepGlobe dataset. The existing single-branch networks mostly use downsampling or chunking to process high-resolution remote sensing images. Therefore, for single-branch networks, we studied the patch inference and global inference sections. Experimental results are shown in Table 3. It can be seen from Table 3 that although Global form can improve segmentation accuracy, it will increase the GPU memory usage. GLNet* refers to the model without feature sharing module. It was found that the mIoU values of common semantic segmentation networks were generally smaller than 65% in the patch inference, and the mIoU values of U-Net [8] and ICNet [55] were smaller than 40%, the memory values were around 1200 MB, and the memory value of FCN-8s was 1963 MB. In the global inference, the mIoU values of various networks were largely similar to those in the patch inference, but the memory values were greatly different from those in the patch inference, and the memory value of SegNet [9] even reached 10,339 MB. Owing to the mode in which the global and local branches collaborate, our Fast-GLNet’s mIoU value reached 72.1%, and its memory value was 1639 MB. In addition, leveraging our DFPM module, Fast-GLNet achieved a superior accuracy, outperforming other comparable methods. As can be seen, the mIoU value of our Fast-GLNet was 0.5% higher than that of GLNet, 0.3% higher than that of PointRend, and 0.2% higher than that of MagNet-Fast. Moreover, Fast-GLNet used less GPU memory than GLNet.

### 4.4. Ablation Study

#### 4.4.1. Multiple Branches

Fast-GLNet processes high-resolution remote sensing images through global and local branches. The results of the ablation study in Table 4 show that double-branch networks are superior to other multibranch networks (networks with three or four branches) in terms of mIoU and memory. The main reason is that, if an excessively large number of branches is used in a semantic segmentation network, too much irrelevant information will be introduced, and excessive noise will affect the network’s capability for semantic segmentation.

#### 4.4.2. Different Fusion Modules

As illustrated in Table 5, the mIoU value of our fusion method reached 72.1, while the mIoU values of the common fusion methods, the Add mode, and the Concate mode were smaller than 70, and the mIoU value for the Add mode was only 55.2. In our DFPA module, we used a feature pyramid similar to an FPN to connect the feature maps from shallow and deep layers, and an aggregation mechanism was incorporated into the encoding part to better merge the features of the two branches and enhance the semantic segmentation network’s prediction performance.

#### 4.4.3. Visualization Result

**Figure 4:**
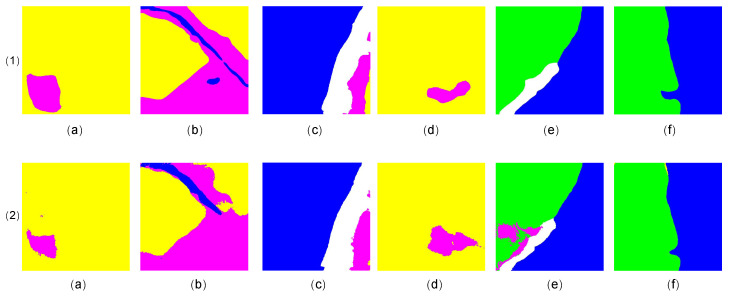
The comparison of segmentation visualization results between our Fast-GLNet and GLNet. Line 1 is the results of Fast-GLNet and Line 2 is the results of GLNet.

**Figure 5:**
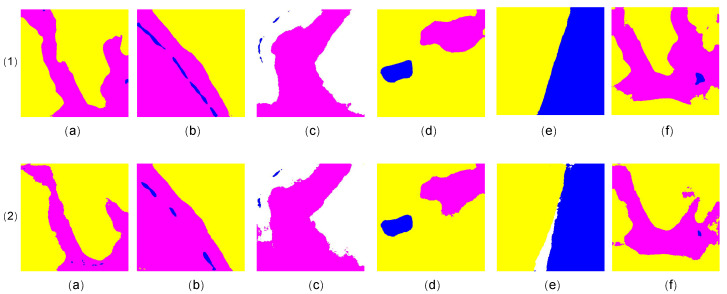
The comparison of segmentation visualization results between our Fast-GLNet and GLNet. Line 1 is the results of Fast-GLNet and Line 2 is the results of GLNet.

In Figure 4 and Figure 5, the segmentation results achieved by Fast-GLNet (line 1) and GLNet (line 2) are compared visually. It can be seen that there were many imprecise boundaries (Figure 4(2(a,d,f)) and broken parts (Figure 4(2(b,c,e)) in the segmentation results of GLNet. By optimizing the feature fusion mode of the global and local branches, Fast-GLNet successfully solved the problems of imprecise boundaries and fragmentation and improved the final quality of the segmentation results.

**Figure 6:**
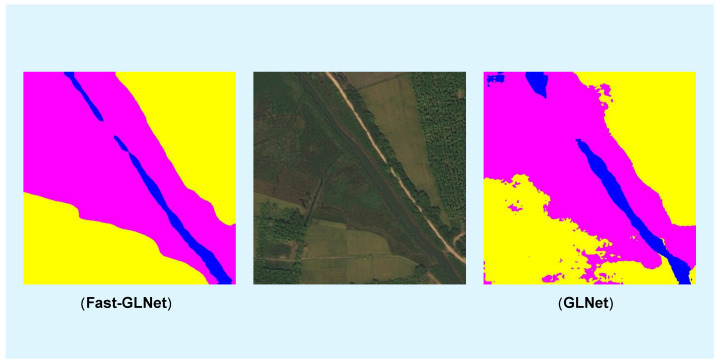
The comparison of segmentation visualization results between our Fast-GLNet and GLNet.

**Figure 7:**
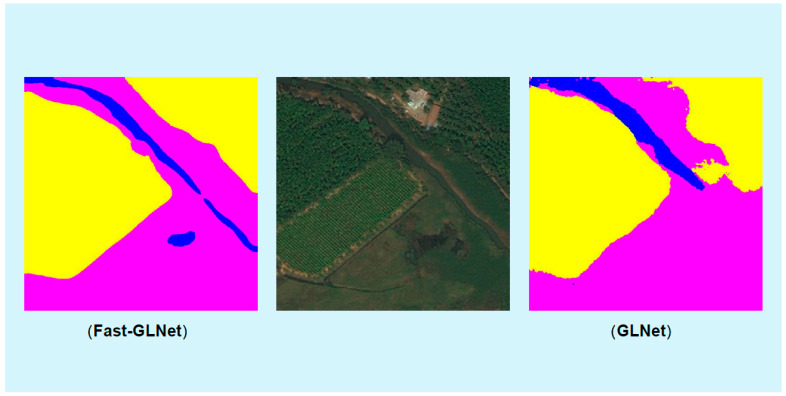
The comparison of segmentation visualization results between our Fast-GLNet and GLNet.

By comparing the segmentation results of the two models in Figure 6 and Figure 7, we can clearly see that there were many small broken parts in GLNet [28] segmentation, which will affect the determination of the boundary. In contrast, our Fast-GLNet improved the segmentation effect at the edge of objects. It has better segmentation accuracy.

## 5. Discussion

### 5.1. Principal Findings and Comparison with Other Methods

Above all, the experiments in this article show that the global and local branch dual-branch structures we chose are superior to single-branch structures and other multibranch structures. In addition, the fusion module we adopted has a higher accuracy than the Add and Concate modes. In addition, in the dual-branch structure network, our Fast-GLNet network structure is superior to most existing dual-branch structure semantic segmentation networks.

We selected some common semantic segmentation networks for horizontal comparison with our Fast-GLNet network. Firstly, we used two common remote sensing image processing datasets, Vaihingen and Postdam. The semantic segmentation networks we selected can be divided into two categories: the single-branch type and the multibranch type. Mean-F1 and mIoU were used as evaluation indexes to compare with mainstream semantic segmentation networks (such as U-Net [8], PsPNet [10], etc.). For example, the mIoU and Mean-F1 of U-Net on the Postdam dataset were 73.9% and 83.9%, while our Fast-GLNet exhibited a better performance, with the mIoU and Mean-F1 being higher at 77.9% and 86.1%. Our Fast-GLNet performance on Postdam and Vaihingen demonstrates its better segmentation accuracy for remote sensing images.

In order to compare the usage of GPU memory, we used the Deepglobal dataset and selected some mainstream single-branch networks and multibranch networks for experiments. In addition, we also compared the segmentation accuracy using the mIoU metric, ensuring that the optimization of GPU memory did not weaken the segmentation accuracy. The experimental results show that, compared to other single-branch and multibranch networks, our Fast-GLNet not only reduces the use of GPU memory but also optimizes the accuracy of semantic segmentation. The existing single-branch networks mostly use downsampling or chunking to process high-resolution remote sensing images. Therefore, for single-branch networks, we studied the patch inference and global inference sections. We found that the GPU memory consumption of all networks in the experiment was positively correlated with segmentation accuracy. When using patch inference, the model consumed lower GPU memory but also had a lower segmentation accuracy. When using global inference, the segmentation accuracy of the model greatly improved, but, at the same time, the consumption of GPU memory also greatly increased. For example, when FCN-8s adopted global conference, the segmentation accuracy greatly improved, with the mIoU increasing from 64.3% to 70.1%, a full 5.8% increase. However, at the same time, the improvement in accuracy was accompanied by a significant increase in GPU memory consumption, which increased from 1963 MB to 5227 MB, an increase of a full 3264 MB. The same situation was also evident on ICNet. After adopting global inference, ICNet’s mIoU increased by 4.7%, from 35.5% to 40.2%. However, at the same time, the GPU memory consumption also increased by 1362 MB, from 1195 MB to 2557 MB. This means that most existing semantic segmentation networks find it difficult to balance the efficiency and accuracy of semantic segmentation. Our dual-branch structure can effectively solve this problem, without sacrificing GPU memory for a higher segmentation accuracy. We can improve segmentation accuracy while balancing segmentation efficiency. In addition, we selected some relatively new multibranch semantic segmentation networks to compare with our Fast-GLNet. These networks included GLNet, CascadePSP [31], PointRend [49], and MagNet-Fast [33]. Thanks to our optimization of feature extraction and fusion, the experimental results show that our network used less GPU memory while achieving a higher segmentation accuracy. For example, GLNet performed at 71.6% mIoU on the Deepglobe dataset and consumed 1865 MB of GPU memory. We proposed a DFPA module on the basis of GLNet, which improved the segmentation efficiency and reduced GPU memory consumption from 1865 MB to 1639 MB, a full 226 MB reduction. Moreover, it also improved the segmentation accuracy and increased the mIoU from 71.6% to 72.1%. That is an increase of 0.5%. This shows that our Fast-GLNet can reduce GPU memory consumption and improve the efficiency of segmentation under the condition of good segmentation accuracy.

In order to more intuitively highlight the improvement of our segmentation accuracy, we visually display the segmentation results of remote sensing images of Fast-GLNet and GLNet. It can be seen that there were many broken and inaccurate parts in the segmentation results of GLNet, which made it difficult to determine the segmentation boundary accurately. However, our Fast-GLNet produced a more accurate segmentation situation, eliminated the broken parts in the segmentation process, had a more accurate boundary, and exhibited a better segmentation accuracy.

In order to demonstrate the advantages of two-branch networks over other branch number networks, we performed an ablation study on different numbers of branches. The experimental results show that, with the increase in the number of branches, the mIoU gradually decreased and GPU memory consumption gradually increased. When the number of branches was two, the mIoU was 72.1%. When the number of branches changed from two to three, the mIoU decreased by 1.7%, from 72.1% to 70.4%. When the number of branches continued to increase to four, the mIoU decreased to 68.4%, which was 3.7% lower than that of the two branches. The GPU memory usage also increased with the number of branches. When the number of branches was two, the GPU memory consumption was 1639 MB. When the number of branches reached three and four, GPU memory consumption increased by 726 MB and 1676 MB, respectively. The ablation study shows that, compared with three-branch and four-branch feature extraction networks, two-branch feature extraction networks avoid the introduction of too much irrelevant information, and have a higher mIoU and better GPU memory utilization efficiency. An excessive number of branches will bring excessive irrelevant information, which will lead to a reduction in segmentation accuracy. In addition, an excessive number of branches will occupy the GPU and consume too much GPU memory, which reduces the efficiency of segmentation. Therefore, the dual-branch structure is a better network structure. Moreover, the dual-branch structure solves the problem of local detail loss or global context information loss in the single-branch structure. This is why the dual-branch structure has a better segmentation accuracy than the single-branch structure.

In addition, the DFPA module similar to FPN [30] designed by us can achieve a better feature fusion effect. We also compared different feature fusion methods. Experiments show that our fusion mode has a higher mIoU. Compared with the Concate and Add fusion modes, the mIoU of our fusion mode was 16.9% higher than that of the Add mode and 4.3% that of the Concate mode. Our Fast-GLNet exhibited a stronger feature fusion capability and can effectively acquire high and low layer features, so as to have a higher accuracy.

Therefore, our network performed better on the Vahigen and Postdam datasets than other mainstream semantic segmentation networks.

### 5.2. Our Network and Its Strengths

How to obtain useful information from remote sensing images has always been a research hotspot. In recent years, semantic segmentation technology based on deep learning has played an outstanding role in processing high-resolution remote sensing images, which can extract features of ground objects in high-resolution remote sensing images, and perform segmentation and classification. However, traditional semantic segmentation networks are often accompanied by high GPU memory consumption and a low segmentation accuracy.

Based on GLNet [28], we adopt a two-branch feature extraction network. The high-resolution remote sensing image is divided into the global part and the local part. The local branch is composed of small images segmented by the original image, which saves the details of the remote sensing image. The global branch is composed of the image formed after downsampling of the original image, which retains the global information of the remote sensing image. Compared with single-branch networks, two-branch networks can retain both local details and global context information. Compared with the multibranch structure, the two-branch structure can solve the problem of excessive irrelevant information caused by an excessive number of branches, so as to avoid a reduction in the segmentation accuracy and an excessive number of branches occupying the GPU and consuming too much GPU memory. Therefore, the two-branch structure is better than other structures with other branch numbers. Moreover, feature extraction and feature fusion were optimized. The aggregation structure proposed by us can better extract features from remote sensing images and obtain features of the local branch and global branch. In addition, a DFPA module similar to FPN was adopted. Features can be fused better, which is faster and consumes less GPU memory. The experimental results show that our Fast-GLNet has a higher segmentation efficiency and accuracy.

### 5.3. Limitations and Future Research Directions

Despite showing advantages in the experimental results, our network still has certain limitations. The segmentation accuracy and GPU memory efficiency can be further optimized. The detection ability of small targets needs to be improved, and there are still problems with missing detection and inaccurate boundary segmentation. In the future, we plan to focus on achieving a better balance between segmentation accuracy and efficiency. Additionally, more lightweight network architectures are also an important research direction. This will help to improve the overall performance of our network and make it more practical for use in real-world applications.

## 6. Conclusions

This research introduces the Fast-GLNet network, a novel approach for efficient semantic segmentation of high-resolution remote sensing images. Fast-GLNet leverages both global and local information to achieve fast and accurate segmentation results, building upon the foundation of GLNet.

The local branch of Fast-GLNet processes small segmented images extracted from the original image, maintaining the same resolution as the initial image. On the other hand, the global branch handles an image created by downsampling the original image. By incorporating the DFPA module and IFS module for feature extraction and prediction, Fast-GLNet constructs a new semantic segmentation network.

Unlike existing semantic segmentation networks, Fast-GLNet excels in optimizing GPU memory utilization and increasing segmentation speed without compromising efficiency.

The experimental results demonstrated that Fast-GLNet effectively balances recognition accuracy and reasoning efficiency, yielding superior overall performance and meeting the demands for the accurate recognition of remote sensing images. Furthermore, it outperforms existing general-purpose semantic segmentation methods, achieving a favorable trade-off between segmentation accuracy and speed.

The introduction of Fast-GLNet contributes to the field by providing a robust solution for rapid and precise semantic segmentation of high-resolution remote sensing images. Its innovative approach, efficient memory utilization, and improved speed make it a valuable tool for various applications that rely on accurate object recognition in remote sensing data. Future research can explore further enhancements and potential extensions of the Fast-GLNet network to address specific challenges in remote sensing analyses.

## Figures and Tables

**Table 1:** Comparison of results on Vaihingen datasets with cutting-edge research.

Method	mIoU	Mean-F1
PSPNet [10]	65.1	76.8
FCN [7]	64.2	75.9
OCnet(ASP-OC) [44]	65.7	77.4
Deeplabv3+ [36]	64.3	76.0
DAnet [45]	65.3	77.1
CCnet [46]	64.3	75.9
SemanticFPN [47]	66.3	77.6
UPerNet [48]	66.9	78.7
PointRend [49]	65.9	78.1
HRNet-W18 [50]	66.9	78.2
GSCNN [42]	67.7	79.5
SFNet [43]	67.6	78.6
EMANet [51]	65.6	77.7
RANet [52]	66.1	78.2
EncodingNet [53]	65.5	77.4
Denseaspp [54]	64.7	76.4
Fast-GLNet	73.2	82.7

**Table 2:** Compariosn with the cutting-edge results on Postdam datasets.

Method	mIoU	Mean-F1
PSPNet [10]	73.9	83.9
FCN [7]	73.1	83.1
OCnet(ASP-OC) [44]	74.2	84.1
Deeplabv3+ [36]	74.1	83.9
DAnet [45]	74.0	83.9
CCnet [46]	73.8	83.8
SemanticFPN [47]	74.3	84.0
UPerNet [48]	74.3	84.0
PointRend [49]	72.0	82.7
HRNet-W18 [50]	73.4	84.0
GSCNN [42]	73.4	84.1
SFNet [43]	74.3	84.0
EMANet [51]	72.9	83.1
RANet [52]	73.8	83.9
EncodingNet [53]	73.4	83.5
Denseaspp [54]	73.9	83.9
Fast-GLNet	77.9	86.1

**Table 3:** Comparison with the cutting-edge results on DeepGlobe datasets.

Network	Patch Inference		Global Inference	
	Memory	mIoU	Memory	mIoU
ICNet [55]	1195	35.5	2557	40.2
U-Net [8]	949	37.3	5507	38.4
PSPNet [10]	1513	53.3	6289	56.6
SegNet [9]	1139	60.8	10039	61.2
DeepLabv3+ [36]	1279	63.1	3199	63.5
FCN-8s [7]	1963	64.3	5227	70.1
GLNet* [28]	1184	57.3	1184	66.4
	**Memory (MB)**		**mIoU (%)**	
CascadePSP [31]	-		68.5	
DenseCRF [56]	1247		70.3	
DGF [32]	1435		70.4	
GLNet	1865		71.6	
PointRend [49]	1593		71.8	
MagNet-Fast [33]	1559		71.9	
Fast-GLNet	1639		72.1	

**Table 4:** Comparison with the results on different branch numbers.

Branch Number	mIoU	Memory
2	72.1	1639
3	70.4	2365
4	68.4	3315

**Table 5:** Comparison with the results on different methods.

Method	mIoU
our fusion	72.1
add	55.2
concate	67.8

## Data Availability

Not applicable.
